# Home climate and habitat drive ecotypic stress response differences in an invasive grass

**DOI:** 10.1093/aobpla/plaa062

**Published:** 2020-11-24

**Authors:** Vasiliy T Lakoba, Jacob N Barney

**Affiliations:** School of Plant and Environmental Sciences, Virginia Tech, Blacksburg, VA, USA

**Keywords:** Agricultural weeds, climate adaptation, ecotype, invasive plants, plant stress, rapid adaptation

## Abstract

Invasive plants and agricultural weeds are a ubiquitous and ever-expanding threat to biosecurity, biodiversity and ecosystem services. Many of these species are known to succeed through rapid adaptation to biotic and abiotic stress regimes, often in highly disturbed systems. Given the current state of evidence for selection of weedy genotypes via primary physiological stresses like drought, flooding, heat, cold and nutrient deficiency, we posit that adaptation to land management regimes which comprise suites of these stresses can also be expected. To establish this link, we tested adaptation to water and nutrient stresses in five non-agricultural and five agricultural populations of the invader Johnsongrass (*Sorghum halepense*) sampled across a broad range of climates in the USA. We subjected seedlings from each population to factorial drought and nutrient stresses in a common garden greenhouse experiment. Agricultural and non-agricultural ecotypes did not respond differently to experimentally applied stresses. However, non-agricultural populations from more drought-prone and nutrient-poor locations outperformed their agricultural counterparts in shoot allocation and chlorophyll production, respectively. We also found evidence for root allocation adaptation to hotter climates, in line with other C4 grasses, while greater adaptation to drought treatment was associated with soil organic carbon (SOC)-rich habitats. These findings imply that adaptation to land-use types can interact with other macrohabitat parameters, which will be fluctuating in a changing climate and resource-needy world. We see that invasive plants are poised to take on novel habitats within their introduced ranges, leading to complications in the prevention and management of their spread.

## Teaser

Johnsongrass, a perennial invader on six continents, is established in both agricultural and non-agricultural habitats across the USA. By testing drought and nutrient stress response of seedlings from populations across the US range, we found that precipitation and soil fertility from the site of population origin mediated drought adaptation, while the latter also impacted differential chlorophyll content of agricultural and non-agricultural ecotypes. Our findings suggest simultaneous rapid adaptation to climate, soil fertility and land use in the establishment of this invasive species. Notably, the non-agricultural ecotype, which emerged after intensive management in cropland, showed greater adaptation to nutrient-poor soils.

## Introduction

Populations of widely distributed species experience a diversity of environments across their ranges, often adapting to major differences in climate, resource availability, competition, trophic interactions and management regimes (Espeland 2013; Oduor *et al.* 2016; van Boheemen *et al.* 2019). This often leads to development of considerable intraspecific variation, which can be an indicator of the species’ local adaptive potential on a continental scale as well as its ability to tolerate habitat and climate change over time (Henn *et al.* 2018; Razgour *et al.* 2019).

Local adaptation is the result of spatially heterogeneous selection pressures that give rise to populations with traits not universally present across environmental gradients (Kawecki and Ebert 2004; Leimu and Fischer 2008). Given enough magnitude and duration of such selection, a single species can yield multiple ecotypes that are distinguishable by traits linked with their geographic origin (Leimu and Fischer 2008). In some plants, intraspecific trait divergence has been shown to correlate with microclimate (Carvajal *et al.* 2017), human land use (Malíková *et al.* 2016) or biotic interactions like herbivory (Garrido *et al.* 2012). Traits linked to divergent ecotypes can include biomass production (Matesanz *et al.* 2012), phenology (Sakaguchi *et al.* 2019), fitness (Armbruster 2014), cold-hardiness (Lowry *et al.* 2014) and pollinator relations (Gervasi and Schiestl 2017). Rapid evolution, local adaptation and ecotypic divergence can readily be seen in invasive plant species which experience a broad spectrum of, often novel, selection pressures across vast ranges (Whitney and Gabler 2008; Oduor *et al.* 2016). In fact, rapid adaptation and ‘de-domestication’ are often seen in feral crop species and manifest across a variety of their traits. A widely studied system exemplifying this is weedy rice (*Oryza sativa* f. *spontanea*), in which Xia *et al.* (2011) found evidence for a change in germination niche adapted to novel ruderal habitats, rather than a reversion to a pre-cultivation phenotype.

Intentional selection is not the only way in which agricultural operations facilitate rapid evolution in weedy species. Rapid evolution of invasive plants has, in some cases, been the consequence of attempts at mechanical and chemical control in agroecosystems (e.g. herbicide resistance in *Amaranthus palmeri*; Salas *et al.* 2016). Some are also exemplars of the role of management in driving local adaptation (Gao *et al.* 2018; Gorton *et al.* 2018). Weedy and invasive plants (i.e. those that exhibit rapid rates of colonization, establishment and spread) have proven to be excellent models for studying adaptation to climate and competition (Engel *et al.* 2011; Atwater *et al*. 2016, 2018). This is, in part, due to the need for considering multiple drivers when studying invasions (Kueffer *et al.* 2013). Abiotic stresses (e.g. climate, drought and nutrient stress) are especially pertinent to invasion biology, where release from biotic and abiotic stressors is fundamental to many invasion hypotheses (te Beest *et al.* 2013; Turner *et al.* 2014). However, little is known about this trend’s extension to subsidies—whether elevated nutrient and moisture levels can drive rapid change in phenotypic responses to different climates. We postulate that this may be the case, given other examples in agricultural systems where irrigation performed as a microclimate creator (Li *et al.* 2008; Cavero *et al.* 2009). Here we test whether populations of the invasive Johnsongrass (*Sorghum halepense*, (L.) Pers. Poaceae) have adapted different stress responses based on their ambient and land use-impacted environments. In this way, we begin to examine whether anthropogenic differences in resource availability are linked with known ecotypes of an invader differing in their response to climatic and edaphic stressors.

Johnsongrass is a perennial grass that is native to parts of the Middle East and is currently a widespread invader in both agricultural and non-agricultural systems worldwide (Warwick and Black 1983). The early introductions of Johnsongrass into North America—in South Carolina in the 1820s and later in Arizona—were into agricultural landscapes as a forage crop (McWhorter 1971), though it quickly became weedy and was abandoned as a crop. The early invasion syndrome was observed in agroecosystems, which frequently led to forced abandonment of lands where mechanical removal of Johnsongrass failed (Ball 1902). Johnsongrass populations are mostly found in the southern half of the contiguous USA, with relatively few pioneer populations in the Northeast, Upper Midwest and Pacific Northwest. Over the course of its nearly 200-year residence in the USA, Johnsongrass has more recently become predominantly an invader of roadside, waste and other non-agricultural spaces (Sezen *et al.* 2016). This shift may have been largely driven by chemical control of Johnsongrass in herbicide-tolerant cropping systems in the late 20th century (Sezen *et al.* 2016).

Across its vast introduced range, Johnsongrass exhibits dramatic genetic and phenotypic variation (Atwater *et al.* 2016; Sezen *et al.* 2016). While some of its success across diverse habitats may be explained by phenotypic plasticity, there is reason to believe that different selection pressures among habitats can manifest over time as ecotypic differentiation (Delêtre *et al.* 2017; Rajakaruna 2018). Such effects have been observed in a range of invasive plants occupying broad novel ranges (Lavergne *et al.* 2010; Molina-Montenegro *et al.* 2018). Previous studies of intraspecific variation in Johnsongrass have largely focused on climatic and large-scale geographic origin as predictors of performance (McWhorter 1971; Burt 1974). Stimulated by the discovery of genomic patterns over the course of the invasion (Sezen *et al.* 2016), recent work has examined the differences in stress and competition response between agricultural and non-agricultural populations (Atwater *et al.* 2018). The findings of differential response based on ecotype identity (i.e. agricultural and non-agricultural) alone are important to the larger question of whether the species is predisposed to generalist adaptability or specialization (Atwater *et al.* 2016).

Given the observed phenotypic differences in Johnsongrass from agricultural and non-agricultural populations and associated population genetic differences (Atwater *et al*. 2016, 2018; Sezen *et al.* 2016), our objective was to test whether ecotypic origin (i.e. agricultural vs. non-agricultural) and environmental selection pressures would predict biomass accumulation and allocation in a common greenhouse experiment during seedling establishment—the most critical stage in population establishment and invasion (Leguizamón *et al.* 2011; Postma and Ågren 2016). Specifically, we were interested in the following questions: (i) Do Johnsongrass home climate and soil fertility drive differences in drought and nutrient stress tolerance? and (ii) Do Johnsongrass ecotypes vary in their drought and nutrient stress tolerance? Results of this study will be useful in elucidating relative degrees of local adaptation to climatic and habitat types across a large and expanding introduced range of this widespread and advancing invader. More broadly, it would be an uncommon head-to-head comparison of different drivers of local adaptation, while most studies focus on a single driver (e.g. climate, elevation, etc.), possibly addressing important interactive effects on invasive species range expansion.

## Methods

### Population selection

**Figure 1.**
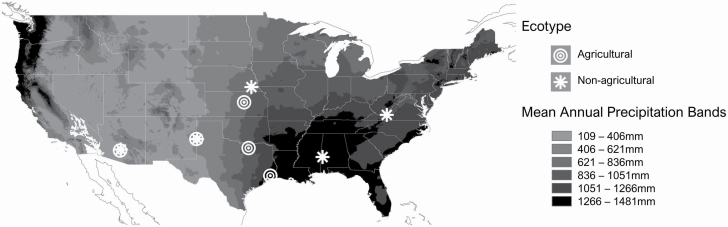
The geographic locations in the contiguous USA where Johnsongrass seed for the experiment was collected. Circles and stars indicate ecotype attribution (agricultural vs. non-agricultural). The shading of the background map corresponds to categorical bands of MAP, with one agricultural and one non-agricultural population sampled from five of the six bands.

We drew from our collection of >200 Johnsongrass populations representing the full geographic and climatic variation of its US range. For this study, we chose from 30 populations that spanned this range, and had been grown for a generation in a common garden to reduce maternal effects, focusing on full representation of the range of mean precipitation as we are examining drought tolerance. We split the set of 30 populations into those collected from agricultural (8) and non-agricultural (22) sites. All populations were plotted in geographic space using ArcMap 10.5.1 (ArcGIS Desktop: Release 10. Redlands, CA: Environmental Systems Research Institute.) overlaid with a 30-year mean annual precipitation (MAP) raster data obtained from PRISM Climate Group, Oregon State University (PRISM Climate Group 2004). The MAP values for the non-agricultural populations ranged from 199 to 1430 mm, while MAP for the agricultural populations ranged from 190 to 1481 mm. These were categorized into six ‘precipitation bands’ of equal range: (1) 190–406 mm, (2) 406–621 mm, (3) 621–836 mm, (4) 836–1051 mm, (5) 1051–1266 mm and (6) 1266–1481 mm. We had viable seed of agricultural and non-agricultural populations in bands 1, 2, 3, 4 and 6. Thus, we arrived at five agricultural populations and five non-agricultural populations which represent the breadth of the precipitation spectrum on which Johnsongrass occurs in the USA (see Fig. 1).

### Plant establishment

To release seeds from dormancy, we treated them with commercial strength sodium hypochlorite (Clorox Regular-Bleach, The Clorox Company, Oakland, CA, USA) for 4 h followed by a 1-h water rinse (Atwater *et al.* 2018). The prepared seeds were then placed in Petri dishes (one dish per population) with four saturated sheets of filter paper (Whatman 1003-055, GE Healthcare, Chicago, IL, USA). Upon radicle emergence, seeds were transferred to 6.4-cm diameter, 656-mL tree pots (Stuewe & Sons, Inc., Tangent, OR, USA) filled with a 2:1 mixture of washed sand to sifted mineral soil collected locally and allowed to establish for 8 days. Individuals from each population were then randomly assigned to one of four treatments: + fertilizer/+ irrigation; + fertilizer/− irrigation; − fertilizer/+ irrigation; and − fertilizer/− irrigation. Pots were arranged in a randomized complete block design with five replicates, with each block representing a greenhouse bench. Irrigation was applied as 10 mL of water daily, keeping the soil near field capacity. Fertilizer (PowerPak 20-20-20, Southern Agricultural Insecticides Inc., Hendersonville, NC, USA) was applied once at time of transplant as a solute in 10 mL of water at a rate of 4 mL L^−1^, which matches the recommended product rate for spray and drench applications. Plants not receiving fertilizer were given 10 mL of water at time of transplant. Due to presumed nutrient availability in the mineral soil which made up one-third of our growing media, the ‘− fertilizer’ treatment more likely created a nutrient limitation than a deficiency. The drought treatments (− irrigation) were administered by reducing irrigation to 5 mL per day on day 1 of treatment, and 0 mL irrigation after 7 days of treatment.

### Measurements

Immediately preceding treatment application, we recorded height to the most recently emerged leaf collar and the number of leaves to account for size asymmetries. Following treatment application, we recorded plant survival, plant height, leaf number, number of culms, chlorophyll content (Chl) and chlorophyll fluorescence ratio (CFR) every 7 days. From Gitelson *et al.* (1999), the formula for chlorophyll content in mg m^−2^ is

$$\begin{array}{l} Chl=634\;\times \;F735/F700-391 \end{array}$$

Chlorophyll fluorescence ratio is the quotient in this equation, or ‘the ratio of fluorescence or fluorescence emission at 735 nm/700 nm’. The latter two were measured using CCM-300 Chlorophyll Content Meter (OPTI-SCIENCES, Hudson, NH, USA). The experiment continued until the most vigorous plants filled their pots with roots, which occurred 28 days after treatment application. At the end of the experiment, presence of rhizome(s) was recorded, and biomass was harvested separately for below-ground and above-ground portions and dried at 75 °C for 7 days.

### Statistical analyses

To describe climate at the place of origin of each population (hereafter ‘home climate’), we used MAP and mean annual temperature (MAT) (PRISM Climate Group 2004), as well as the soil organic carbon content (SOC) (Hengl *et al.* 2014) as a descriptor of habitat nutrient availability. We chose SOC to represent soil fertility (or nutrient availability) based on findings of strong positive relationships between SOC and N/P/K availability in agroecosystems (Cheng *et al.* 2016). Following analysis of response variable correlation, we used three independent response variables: total biomass, CFR and root-to-shoot biomass ratio. The total biomass and root:shoot variables were log_10_-transformed to meet assumptions of the linear model. Each response variable was analysed in a mixed effects linear model with block as a random effect and height-at-transplant, MAP, MAT and SOC as fixed effects, and ecotype (agricultural vs. non-agricultural), fertilization and irrigation as fixed effects, as well as all possible second-order interactions. We then performed backwards model selection, removing non-significant predictors to minimize corrected Akaike Information Criterion. The main effects of block, initial height, irrigation treatment and fertilization treatment were kept in all models. All statistical analyses were performed using JMP Pro, Version 15 (SAS Institute, Inc., Cary, NC, USA).

## Results

### Total biomass, root:shoot and chlorophyll content are correlated with irrigation and/or fertilization

**Figure 2.**
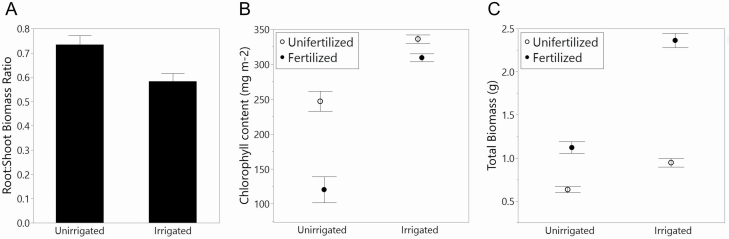
(A) The effect (mean ± SE) of irrigation treatment on root:shoot ratio (*P* = 0.0008). The interactive effect (mean ± SE) of irrigation and fertilization on (B) chlorophyll content (*P* < 0.0001) and (C) total biomass (*P* < 0.0001).

The irrigation and fertilization treatments had strong positive interactive effects on total biomass as well as chlorophyll content (Table 1). Fertilization resulted in a 59 % increase in biomass among irrigated individuals (0.96–2.35 g), but only a 44 % increase among unirrigated individuals (0.61–1.1 g) (Fig. 2C). Leaf chlorophyll content increased by only 9 % due to fertilization among irrigated plants (306–338 mg m^−2^), while fertilization of unirrigated plants yielded a 51 % increase in leaf chlorophyll content (122–249 mg m^−2^) (Fig. 2B). Mean root:shoot of unirrigated populations (0.7) was significantly higher than that of irrigated populations (0.5) (Fig. 2A).

**Table 1.** Effect tests of each linear model as reduced via backward stepwise selection for optimized AICc. Only effects that remained in at least one of the three reduced models are presented in this table. Total biomass and root:shoot response variables were log-transformed to meet model assumptions. Square brackets around variable names indicate variable locking prior to stepwise selection. Alpha level of significance indicated by *** = 0.0005, ** = 0.005, * = 0.05.

	Total biomass				Root:shoot				Chlorophyll			
	Degrees of freedom	Sum of squares	*F*	*P*	Degrees of freedom	Sum of squares	*F*	*P*	Degrees of freedom	Sum of squares	*F*	*P*
[Block]	4	0.201	8.655	0.0037**	4	0.046	1.287	0.258	4	2108.2	0.278	0.5993
[Initial height]	1	1.325	57.11	<0.0001***	1	0.02	0.554	0.458	1	21 735	2.856	0.0927
[Irrigation]	1	0.662	28.54	<0.0001***	1	0.41	11.57	0.0008**	1	204 010	26.81	<0.0001***
[Fertilization]	1	1.647	70.99	<0.0001***	1	0.091	2.574	0.1103	1	407 796	53.59	<0.0001***
Ecotype	–	–	–	–	–	–	–	–	1	1987.7	0.261	0.6099
MAT	–	–	–	–	1	0.302	8.618	0.0037**	–	–	–	–
MAP	–	–	–	–	1	0.062	1.759	0.1863	–	–	–	–
SOC	–	–	–	–	–	–	–	–	1	57 303	7.5306	0.0067*
Irrigation * Fertilization	1	1.181	50.92	<0.0001***	–	–	–	–	1	121 443	15.96	<0.0001***
Irrigation * MAP	–	–	–	–	1	0.215	6.065	0.0147*	–	–	–	–
Irrigation * SOC	–	–	–	–	–	–	–	–	1	33 967	4.464	0.0359*
Ecotype * SOC	–	–	–	–	–	–	–	–	1	32 704	4.298	0.0395*

### Chlorophyll content response to SOC in home habitat is mediated by ecotype identity

**Figure 3.**
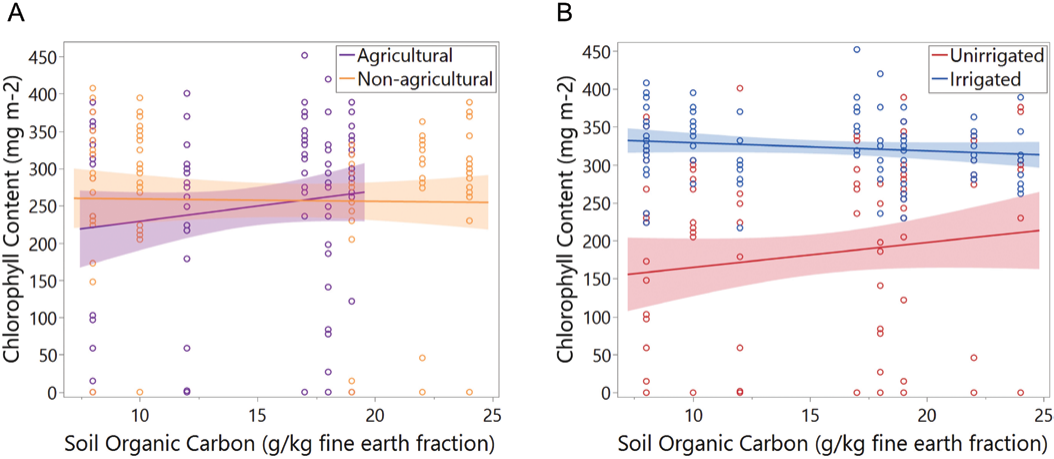
The interactive effect of (A) ecotype identity and SOC in home habitat on chlorophyll content (*P* = 0.0395). The interactive effect of (B) irrigation treatment and SOC in home habitat (*P* = 0.0359). Error bands in both panels represent 95% C.I.

Leaf chlorophyll content varied between ecotypes as a function of SOC in their home habitat (Fig. 3A). Non-agricultural populations from habitats across SOC levels had nearly uniform chlorophyll production. Agricultural populations, however, produced significantly more chlorophyll in populations from home habitats of high SOC. Among populations from the most SOC-poor origins of the USA, non-agricultural populations contained as much as 50 mg m^−2^ more chlorophyll than agricultural populations. Among populations whose home habitats had SOC of >17 g kg^−1^, agricultural populations overtook non-agricultural counterparts in chlorophyll production.

### Chlorophyll content and root:shoot biomass ratio respond to home habitat parameters

**Figure 4.**
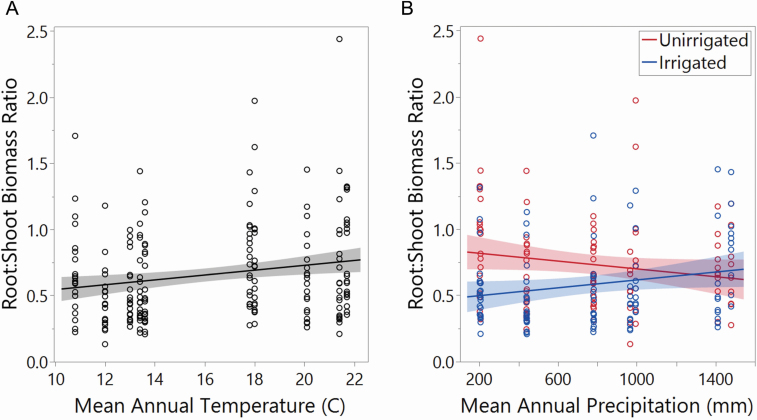
The effect of (A) MAT in home habitat on root:shoot biomass ratio (*P* = 0.0037). The interactive effect of (B) irrigation and MAP at home habitat on root:shoot biomass ratio (*P* = 0.0147). Error bands in both panels represent 95 % CI.

Johnsongrass population differences in root:shoot ratio were affected by home climate, while chlorophyll content differences responded to SOC. We observed a mild, yet significant, positive relationship between home habitat MAT and Johnsongrass root:shoot biomass ratio across populations (Fig. 4A). An increase in root:shoot ratio from 0.6 to 0.78 was associated with an increase in home MAT from 11 to 22 °C, which did not vary between ecotypes.

Root:shoot ratio response of irrigated and unirrigated plants differed based on home habitat MAP (Fig. 4B). The root:shoot ratio of Johnsongrass grown with non-limiting soil moisture increased with higher MAP, while we observed a decreasing root:shoot ratio in droughted Johnsongrass as MAP increased (*P* = 0.0147). In other words, drought produced no significant difference in root:shoot biomass ratio (R:S) among plants from the highest precipitation home habitats, while leading to a significant increase (from an average of 0.49 to 0.78) in R:S among plants from the most arid origins.

While Johnsongrass home habitat SOC had a slightly negative effect on chlorophyll content of irrigated plants, it had a positive effect on chlorophyll content in unirrigated plants (see Fig. 3B). Thus, the irrigation treatment was responsible for a 67 % greater difference in chlorophyll content between samples from SOC-poor habitats (180 mg m^−2^, on average) than on those from SOC-rich habitats (120 mg m^−2^, on average).

## Discussion

We found evidence for local adaptation in Johnsongrass to home habitat parameters (MAT, MAP and SOC) as well as differential ecotypic adaptation. Responses to home habitat and ecotype identity interacted with experimentally applied drought and nutrient stresses. Mean annual temperature was the only home habitat variable found to impact seedling performance (specifically root allocation) on its own, while MAP, SOC and ecotype identity had interactive effects on plant response. Given earlier studies that find Johnsongrass ecotypic differences in competition (Atwater *et al.* 2016), stress response adaptation for competitive advantage (Schwinning *et al.* 2017), as well as abundant intraspecific variation globally (McWhorter 1971), our findings of further local adaptation in various seedling traits provide additional evidence for how this species has become so widespread across a large and diverse geographic area. We can now interpret evidence of ecotypic differences in stress response while controlling for other abiotic habitat qualities. Our results suggest that the two Johnsongrass ecotypes, agricultural and non-agricultural, may have differently adapted to nutrient limitation. Our approach in sampling populations of both agricultural and non-agricultural origins along extensive MAP, MAT and SOC gradients has given us new evidence of Johnsongrass adaptation associated specifically with human land uses. The fact that this effect was mediated by nutrient availability brings us closer to testing its mechanisms in the future. Commonly suggested physiological mechanisms for nutrient stress tolerance in other species include tissue life maximization (Chapin 1987) and microbial symbiosis (Johnson *et al.* 2010). The latter, with regard to N fixation, has been found in some non-agricultural Johnsongrass populations (Rout *et al.* 2013).

There are numerous examples of drought tolerance advantages among invasive plants (e.g. Filippou *et al.* 2014; Vetter *et al.* 2020) and among C4 invaders in particular (Baruch and Jackson 2005; Fahey *et al.* 2018). Drought is one of many stresses which is thought to select for invasive species and communities of invaders over native counterparts (Catford *et al.* 2012). Stochastic stress tolerance in invasive plants is also seen as a key element of their r-selection strategy (Berger and Ludwig 2014). By parsing seedling root and shoot biomass, we were able to examine Johnsongrass seedling drought response in relation to its ecotype identity and home habitat conditions.

Root:shoot biomass ratio is an indicator of plant health, particularly in its extreme values. Ratios approaching zero are indicative of very low root allocation, which signals overall poor health (Thornley 1972; Wilson 1988). Ratios close to 1 (not an absolute rule across species) indicate overall good health. Ratios substantially higher than 1 indicate intensified root allocation, which is a proxy for drought stress response in a vast number of species (Eziz *et al.* 2017). Increase in root:shoot ratio in response to drought stress was also found in our experiment. Eziz *et al.*’s (2017) meta-analysis found that the root allocation response of perennial herbs, of which Johnsongrass is one, is less drought-sensitive than annual herbs, but more sensitive than that of woody plants. This finding suggests that a plant’s drought sensitivity may be directly related to its degree of investment in perenniating tissues. Specifically, highest sensitivity was linked with no such investment (annuals) and lowest sensitivity corresponded to maximal investment (woody plants), while intermediate drought sensitivity was associated with perennial herbaceous plants. Johnsongrass’s rhizomes are critical in its evasion of and resilience to cold stress (Warwick *et al.* 1986; Washburn *et al.* 2013) and could be similarly linked with drought response. However, this could not have been the cause of seedling drought tolerance in our study due to negligible rhizome production at this early life stage. This suggests that a physiological mechanism—perhaps even one inherited from its ancestor *S. bicolor* (Sanchez *et al.* 2002)—may be involved in Johnsongrass’s drought response.

The positive response of root:shoot ratio to Johnsongrass home MAT is potentially linked to the metabolic physiology of Johnsongrass and, indirectly, its success as an invader. Two global meta-analyses—one of 7763 terrestrial ecosystems (Qi *et al.* 2019) and another of >6200 forests (Reich *et al.* 2014)—found that plants growing in colder climates have a higher root:shoot ratio. However, research on *Sorghum bicolor* (Clark and Reinhard 1991) and *Cleistogenes* spp. (Luo *et al.* 2013), both C4 grasses like Johnsongrass, shows root:shoot ratio increasing with higher MAT. To explain this, Luo *et al.* (2013) cite general C-allocation advantages of C4 over C3 plants under high CO_2_ and high temperature/aridity conditions (Ehleringer *et al.* 1997). While our study only examined intraspecific variation in a C4 grass, rather than comparing it with C3 species, it stands to reason that the positive relationship between MAT and Johnsongrass root allocation was indicative of adaptation to hotter and drier home habitats. This finding poses the question of whether Johnsongrass’s heightened root allocation impacts its competitive—and therefore invasive—ability. Johnsongrass is known to compete with crops (e.g. maize) largely below-ground (Acciaresi and Guiamet 2010), which supports the idea that its higher C4-facilitated performance in hotter habitats may translate into a competitive advantage. However, its below-ground competitive effect is difficult to test empirically in complex plant communities (Schwinning *et al.* 2017).

Populations from arid climates responded most strongly to the difference in water availability by allocating significantly more to roots under drought conditions. Compared to mesic home habitat populations, which showed no change in root allocation during drought, arid origin populations showed an important plastic response. This suggests that frequent drought stress may have selected for this plastic response in Johnsongrass populations from arid regions, but not their counterparts from places where drought is rare. Studies on other C4 grasses suggest that variation in drought response adaptations may be indicative of the type or duration of drought associated with selection in home habitats. For example, Cardoso *et al.* (2015) compared drought response in two C4 grasses and found a suite of responses, including plastic increase in root:shoot ratio to be associated with the species that had adapted to intermittent drought, rather than prolonged drought. From the perspective of deep evolutionary time, Zhou *et al.* (2018) assert that water limitation (given high atmospheric CO_2_) was the primary driver of C4 evolution, while today its advantage over C3 is often associated with elevated temperatures (Ehleringer *et al.* 1997). In a Texas grassland community context, Wilsey and Polley (2006) found both C3 and C4 grasses (Johnsongrass not among them) to respond plastically to drought; however, native grasses had a greater increase in root:shoot ratio than invaders. Schwinning *et al.* (2017)—while unable to compare below-ground allocation—also found that Johnsongrass seedlings’ advantage over native competitors was significant above-ground. Thus, given contextual evidence to date, root:shoot plasticity in Johnsongrass from arid, but not mesic, regions is more likely an adaptation linked with general performance in a stressful growing environment rather than a competitive advantage.

We saw that SOC-poor origin populations experienced greater differences in chlorophyll content due to irrigation/drought than their SOC-rich origin counterparts. This suggests that habitats with higher nutrient availability may release plants from stresses limiting chlorophyll production, and this is less impacted by drought than in plants from low-SOC habitats. We saw that the irrigation treatment’s interaction with SOC in home habitat mirrored its interaction with the fertilization treatment. In the latter case, the impacts of nutrient limitation were significantly lowered by irrigation. Had we been able to grow the ‘− fertilizer’ plants in nutrient-free media, it is more than likely that this relationship would have been conserved, if not strengthened. Considering that agricultural populations increase chlorophyll content with the home SOC gradient, while non-agricultural populations do not, there may be a parallel between drought response and ecotypic adaptation. Specifically, we saw that, with increasing home SOC, unirrigated and agricultural populations produce more chlorophyll, while the chlorophyll content of irrigated or non-agricultural populations does not change. While we did not find a direct interaction between ecotype identity and the irrigation treatment, the combination of our findings suggests it. Speculatively, the agricultural ecotype’s response to home SOC may reflect higher stress due to less drought adaptation. Meanwhile, the non-agricultural ecotype shows similar unresponsiveness to home SOC as all unirrigated populations—a condition to which it may be adapted. The interactive effect of irrigation and fertilization on chlorophyll content has been seen in wheat (Dimkpa *et al.* 2020), while in *S. bicolor* it may be mediated by microbes aiding in phosphorus acquisition (Kour *et al.* 2020). Others have found instances of wild and cultivated ecotypes with differentially adapted nutrient uptake and chlorophyll production (Papafilippaki and Nikolaidis 2020). Kausar and Gull (2019) found some genotypes of *S. bicolor* to be more tolerant of salinity stress (which inhibited normal nutrient uptake) than others. In fact, much of the research that compares plant ecotypic differences in chlorophyll content does so in the context of tolerance to polluted soils and their phytoremediation (Park *et al.* 2012; Alirzayeva *et al.* 2017; Liu *et al.* 2019). Our comparison of home habitat and ecotype identity as drivers of adaptation is novel; however, our approach yielded results similar to others that found ecotypic differences in experimental stress response.

Population differences in stress response at the seedling stages suggest advantages for producing founder populations in new locations. The capacity for these stress adaptations in invasive plants has often been associated with greater spread and persistence (Wen 2015; Yuan and Wen 2018; Abbas *et al.* 2019). Evolutionary change concurrent with landscape spread has also been linked with higher fecundity and ability to occupy new habitats (Lustenhouwer *et al.* 2019). We see that no life stage is alone responsible for invasion success, but rather a suite of mechanisms interacts over time to boost the invader’s fitness. Studying seedlings is advantageous for discerning the roles of abiotic stresses to which plants later become less vulnerable, while limiting experiments to this stage may inhibit our ability to understand the invader’s competitive ability, which is dynamic over time. As our interests here were limited to the former, the seedling experiment was most appropriate.

Overall, we found support for differences in drought stress based on home habitat properties—namely, water availability (MAP) and soil fertility (SOC). We did not find evidence for differences in nutrient stress response based on home habitat properties MAT, MAP or SOC. We found ecotype identity to moderate performance differences based on SOC in home habitat; however, we found no direct relationships between ecotype and MAT, MAP, or the irrigation and/or fertilization treatments. We can use these interpretations to reflect more broadly on long-term trajectories of biological invasions. Human landscape management across fine and coarse scales can have an enormous impact on the distribution (Decker *et al.* 2012) and adaptation of invaders (Schneider 2006). At the same time, selection for plasticity and ‘plasticity of plasticity’ may be a common trait among invasive species (Funk 2008; Davidson *et al.* 2011). With land-use change and accompanying changes in management regimes in disturbed ecosystems selecting for adaptable lineages, we expect to see similar patterns across taxa. Numerous studies have found nutrient pollution (Crooks *et al.* 2011), heavy metal pollution (Yang *et al.* 2007) and urban heat island effect (Battles and Kolbe 2019) to facilitate invasion through the creation of suitable microhabitats, sometimes to the exclusion of other competitors. In particular, landscape novelty and heterogeneity in urbanized areas present great challenges to our understanding of invader adaptation and potential management (Gaertner *et al.* 2017). In parallel with this work, it will be important to investigate whether selection through land-use change has the potential to impact ecotypes’ adaptation to climate change and other habitat modifications.

## Supplementary Material

plaa062_suppl_Supplementary_Data

## Notes

**Form & Function. Chief Editor:** Kate McCulloh
